# Cognitive Impairment Following Acute Mild Traumatic Brain Injury

**DOI:** 10.3389/fneur.2019.00198

**Published:** 2019-03-08

**Authors:** Maíra Glória de Freitas Cardoso, Rodrigo Moreira Faleiro, Jonas Jardim de Paula, Arthur Kummer, Paulo Caramelli, Antônio Lúcio Teixeira, Leonardo Cruz de Souza, Aline Silva Miranda

**Affiliations:** ^1^Neuroscience Program, Laboratório Interdisciplinar em Investigação Médica, Universidade Federal de Minas Gerais, Belo Horizonte, Brazil; ^2^Faculdade de Ciências Médicas de Minas Gerais, Fundação Hospitalar do Estado de Minas Gerais, Belo Horizonte, Brazil; ^3^Faculdade de Ciências Médicas de Minas Gerais, Belo Horizonte, Brazil; ^4^Laboratório Interdisciplinar em Investigação Médica, Eli Lilly and Company do Brasil, São Paulo, Brazil; ^5^Departamento de Clínica Médica, Faculdade de Medicina da Universidade Federal de Minas Gerais, Belo Horizonte, Brazil; ^6^Santa Casa BH Ensino e Pesquisa, Neuropsychiatry Program, Department of Psychiatry and Behavioral Sciences, McGovern Medical School, University of Texas Health Science Center at Houston, Houston, TX, United States; ^7^Laboratório Interdisciplinar em Investigação Médica, Departamento de Clínica Médica, Faculdade de Medicina da Universidade Federal de Minas Gerais, Belo Horizonte, Brazil; ^8^Laboratório Interdisciplinar em Investigação Médica, Faculdade de Medicina da Universidade Federal de Minas Gerais, Belo Horizonte, Brazil; ^9^Laboratório de Neurobiologia, Departamento de Morfologia, Instituto de Ciências Biológicas da Universidade Federal de Minas Gerais, Belo Horizonte, Brazil

**Keywords:** traumatic brain injury, cognitive impairment, loss of consciousness, Brazilian patients, episodic memory

## Abstract

Patients with mild traumatic brain injury (mTBI) may present cognitive deficits within the first 24 h after trauma, herein called “acute phase,” which in turn may lead to long-term functional impairment and decrease in quality of life. Few studies investigated cognition in mTBI patients during the acute phase. The objectives of this study were to investigate the cognitive profile of patients with mTBI during the acute phase, compared to controls and normative data, and whether loss of consciousness (LOC), previous TBI and level of education influence cognition at this stage. Fifty-three patients with mTBI (aged 19–64 years) and 28 healthy controls participated in the study. All patients were evaluated at bedside within 24 h post-injury. Demographic and clinical data were registered. Cognitive function was assessed with the Mini-mental state examination (MMSE), the Frontal Assessment Battery (FAB), Digit Span (working memory), and the Visual Memory Test/Brief Cognitive Battery (for episodic memory). The clinical sample was composed mainly by men (58.5%). The mean age was 39 years-old and 64.3% of the patients had more than 8 years of education. The most common causes of mTBI were fall from own height (28.3%), aggression (24.5%), and fall from variable heights (24.5%). Compared to controls, mTBI patients exhibited significantly worse performance on MMSE, FAB, naming, incidental memory, immediate memory, learning, and delayed recall. Compared to normative data, 26.4% of patients had reduced global cognition as measured by the MMSE. Episodic memory impairment (13.2%) was more frequent than executive dysfunction (9.4%). No significant differences were found in cognitive performance when comparing patients with or without LOC or those with or without history of previous TBI. Patients with lower educational level had higher rates of cognitive impairment (VMT naming−28.6 vs. 4.2%; VMT immediate memory−32 vs. 4.2%; VMT learning−39.3 vs. 4.2%, all *p* < 0.05). In sum, we found significant cognitive impairment in the acute phase of mTBI, which was not associated with LOC or history of TBI, but appeared more frequently in patients with lower educational level.

## Introduction

Traumatic brain injury (TBI) is a highly prevalent condition, affecting men and women of all ages and socioeconomic status worldwide (1, 2). The annual incidence of TBI is estimated in 295 for 100,000 people. Most TBIs occur in males and are mild (1). According to the World Health Organization, mild traumatic brain injury (mTBI) is defined as an acute brain injury resulting from external forces to the head that causes one or more of the following: confusion or disorientation; loss of consciousness (LOC) ≤30 min or other transient neurologic abnormalities; post-traumatic amnesia for <24 h; Glasgow Coma score ranging from 13 to 15 (3).

Patients with mTBI may experience cognitive deficits in the first hours following the trauma (4–7). It has been reported that at 3–5 days after injury, patients with mTBI performed significantly worse compared to orthopedically injured patients and healthy controls in different cognitive tasks, such as immediate recall, short-delayed recall, long-delayed recall, attention, working memory, processing speed and other executive functions (8–10). Importantly, some deficits may be detected even 1 year after the trauma (11).

Features such as loss of consciousness (LOC) or having a previous history of TBI seem to influence cognitive performance in patients with mTBI (12–15). A history of repetitive mTBI was associated with deficits in delayed memory and executive function in a meta-analysis that investigated neuropsychological impact of multiple concussions (15). Previous TBI and LOC were also prognostic markers of persistent post-concussive symptoms after mTBI in deployed-veterans (16).

Although high-quality epidemiological data are scarce in Brazil (17), it has been estimated that there are around 125,000 hospital admissions due to TBI per year, with an incidence of 65.7 admissions per 100,000 inhabitants and a hospital mortality of 5.1/100,000/year (18). Beyond direct costs with hospital expenses, the reliable quantification of the burden caused by TBI is difficult to establish owing to inadequate standardization and incomplete data collection on the incidence and outcome of brain injury.

Only few studies investigated the cognitive status of mTBI patients during the first 24 h post-injury, herein called “acute phase,” and the factors possibly associated with cognitive performance in this early stage (4–7), such as LOC and previous TBI.

In the current study, we aimed to investigate the cognitive performance of patients with acute mTBI, compared to healthy controls and normative data, as well as to address whether LOC or history of TBI influence cognition and whether years of education might present a potential protective role in mTBI-associated cognitive impairment.

## Methods

### Study Design and Participants

The present study was conducted at João XXIII Hospital/Fundação Hospitalar do Estado de Minas Gerais (FHEMIG), a trauma reference center situated in Belo Horizonte, Minas Gerais, Brazil (Southeast), which has more than 500 trauma dedicated beds. In 2015, 1,862 TBI patients were admitted to this Trauma center, with a mortality rate of 34% in cases of severe TBI.

During a period of 30 days, two times a day (at 12 p.m. and 6 p.m.), all patients admitted to the hospital were registered. We included patients with mTBI (scores between 13 and 15 at the Glasgow Coma Scale—GCS (19)—at first evaluation at the emergency department), with < 24 h post-TBI, aged between 18 and 65 years-old, who agreed to participate by providing written informed consent. Patients with epilepsy, history of neurosurgery, dementia, skull fracture, penetrating injury, or any acute intracranial findings on brain scan were excluded. Demographic, clinical and neuropsychological data were obtained.

**Figure 1:**
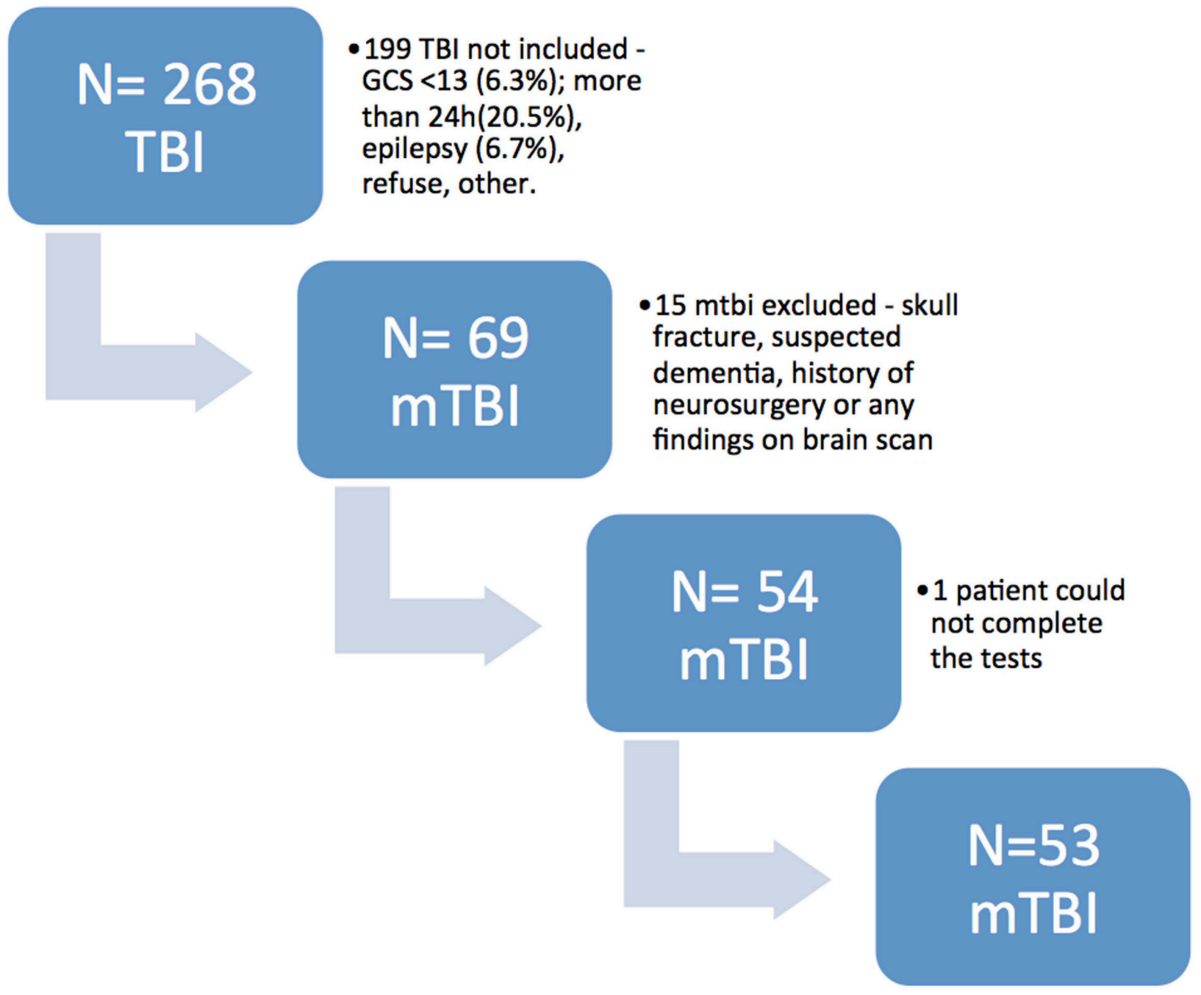
Flow diagram of patient's recruitment. TBI, traumatic brain injury; mTBI, mild traumatic brain injury.

During the study period from November 10th to December 10th, 2016, 268 patients with TBI were admitted to the emergency department. One-hundred and 99 patients did not meet the inclusion criteria. From the 69 patients included, 15 were excluded for having skull fracture, suspected dementia, history of neurosurgery or findings in the brain scan. One patient was excluded because he could not complete the cognitive tasks. The final sample was composed by 53 patients (Figure 1). All patients were assessed at the bedside.

This study was approved by the local ethics committees (protocol number CAAE: 49623015.0.0000.5149) and all patients provided written informed consent prior to study participation.

The control group was composed by 28 healthy participants recruited from the local community, aged between 18 and 65 years-old, who agreed to participate by providing written informed consent. Participants were included if they have no past history of TBI. Exclusion criteria were previous neurosurgery, neurodegenerative diseases, stroke, and epilepsy, other neurological disorders and/or cognitive decline (i.e., delirium or dementia) and significant sensory impairment.

### Clinical Evaluation

All participants answered a sociodemographic and medical questionnaire. Cognitive performance was assessed with the following tests:

#### Mini Mental-State Examination—MMSE (20, 21)

This test evaluates global cognitive status. It includes tasks that evaluate temporal and spatial orientation, memory, attention, naming, following verbal and written commands, writing and copy.

#### Frontal Assessment Battery—FAB (22, 23)

This battery was designed to assess frontal lobe functions which mediate executive functioning. It contains six tasks that assess conceptualization and abstract reasoning, mental flexibility, motor programming, sensitivity to interference, inhibitory control and environmental autonomy.

#### Digit Span Subtest From the Wechsler WAIS-III (24)

This subtest is a number repetition task and is constituted by two tasks—digit span forward and digit span backward—which evaluate attention and working memory.

Episodic memory was assessed with **the Visual Memory Test** from the Brief Cognitive Battery—VMT (25, 26). This latter test consists of ten simple line drawings. Patients are asked to name the drawings and then an incidental recall is required. Drawings are shown again and patients are explicitly informed that they should memorize them. Patients undergo a new recall (immediate recall) and then the procedure is repeated (learning recall). Patients undergo an interference task and a delayed recall (5 min after) is required. The last procedure is a recognition task of the 10 original drawing among 10 distractors.

### Statistical Analysis

Data analyses were performed using the Statistical Package for the Social Sciences (SPSS), version 17 (SPSS Inc., Chicago, IL, USA). Descriptive data were analyzed in frequencies, mean, standard deviation, median and 25 and 75 percentiles. All variables were tested for normal distribution by employing the Shapiro-Wilk test.

Differences of sex distribution and educational level among groups were tested using Pearson Chi-square test for categorical data. Differences of age and neuropsychological performance between groups were tested using Mann-Whitney test. Pearson Chi-square test was used to compare frequencies between groups. Spearman's test was used to explore correlations among sociodemographic, clinical, and neuropsychological data. Cohen's d effect sizes were calculated for comparisons between clinical groups.

Deficit was defined as 2 standard deviations below test's normative data. The normative data provided different means and standard deviations according to the individual's educational level.

All statistical tests were two tailed and a significance level was set at 0.05.

## Results

The final clinical sample was composed of 53 patients with mTBI (Figure 1). Sociodemographic and clinical features are shown in Table 1. The mTBI sample was composed mainly by men (58.5%) with a mean age of 39.1 years, ranging from 19 to 64 years. Most patients (92.5%) had a score of 15 at GCS and 64.3% had 8 or more years of education. The most frequent causes of TBI were fall from own height, accounting for 28.3% of cases, aggression (24.5%) and fall from variable heights (24.5%). Only 7.5% of mTBIs were caused by traffic accidents. Eleven patients (20%) reported alcohol consumption in the previous 12 h.

**Table 1:** Sociodemographic and clinical features of patients with acute mild traumatic brain injury.

**Sex**	***N***	**%**
Male	31	58.5
Female	22	41.5
**MARITAL STATUS**		
Single	30	56.6
Married	22	41.5
Divorced	1	1.9
**YEARS OF EDUCATION**		
< 8 years	19	35.8
8 years	10	18.9
< 11 years	3	5.7
11 years	18	34
>12 years	3	5.7
**SCORE AT GCS**		
GCS 13	1	1.9
GCS 14	3	5.6
GCS 15	49	92.4
**CAUSES OF TBI**		
Fall (from own height)	15	28.3
Aggression	13	24.5
Fall (from variable heights)	13	24.5
Accident with vehicles	5	9.4
Repetitive trauma	4	7.5
Other	3	5.6
**LOSS OF CONSCIOUSNESS**		
Yes	27	50.9
No	25	47.2
Unknown	1	1,9
**PREVIOUS TBI**		
Yes	28	52.8
No	23	43.4
Unknown	2	3,8

*GCS, Glasgow coma scale; TBI, Traumatic brain injury*.

Regarding cognitive performance, mTBI patients presented the following means and standard deviations: MMSE (mean = 23.8, *SD* = 4.1, *n* = 52), FAB (mean = 12.2, *SD* = 2.8, *n* = 53), Visual Memory Test naming (mean = 9.8, *SD* = 0.4, *n* = 53), VMT incidental memory (mean = 5.7, *SD* = 1.3, *n* = 52), VMT immediate recall (mean = 7.7, *SD* = 1.5, *n* = 52), VMT learning (mean = 8.2, *SD* = 1.5, *n* = 52), VMT delayed recall (mean = 7.5, *SD* = 1.7, *n* = 52), VMT recognition (mean = 9.9; *SD* = 0.3, *n* = 52), Digit Span Forward (mean = 4.7, *SD* = 1.0, *n* = 53) and Digit Span Backward (mean = 3.3, *SD* = 1.2, *n* = 53).

The healthy control group was composed by 28 participants aged between 18 and 63 years-old (mean = 39.4, *SD* = 12.8), 50% (*N* = 14) of the sample was composed by men, and 71% had 8 or more years of education. Controls showed the following means and standard deviations on cognitive tests: MMSE (mean = 27.2, *SD* = 2.1), FAB (mean = 13.6, *SD* = 3), Visual Memory Test naming (mean = 10), VMT incidental memory (mean = 7.1, *SD* = 1.5), VMT immediate recall (mean = 8.7, *SD* = 0.9), VMT learning (mean = 9.4, *SD* = 0.9), VMT delayed recall (mean = 9, *SD* = 1.1), VMT recognition (mean = 10), Digit Span Forward (mean = 5.2, *SD* = 1.3), and Digit Span Backward (mean = 3.5, *SD* = 0.7).

Controls had no significant differences from mTBI patients on age (*p* = 0.94), sex (*p* = 0.47), and years of education (*p* = 0.15). Comparison between groups' cognitive performance is shown on Table 2. Patients significantly differed from controls on MMSE (*p* < 0.001), VMT naming (*p* = 0.02), VMT incidental memory (*p* = 0.001), VMT immediate recall (*p* = 0.003), VMT learning (*p* < 0.001), VMT delayed recall (*p* < 0.001), and FAB (*p* = 0.047) scores. There were no significant differences between groups on VMT recognition, Digit Span forward and Digit Span backward scores.

**Table 2:** Cognitive performance comparison between mTBI patients and healthy controls.

**Variables**	**MTBI patients (percentile 25–75)**	**Healthy controls (percentile 25–75)**	**Mann-Whitney test**
*N*	53	28	–
MMSE	25 (22–26.75)	27 (26–29)	***p*** **< 0.001**
FAB	12 (11–14)	14 (10.5–16)	***p*** **= 0.047**
VMT-naming	10 (10–10)	10 (10–10)	***p*** **= 0.020**
VMT-incidental memory	6 (5-7)	7 (6–8.75)	***p*** **= 0.001**
VMT-immediate memory	8 (7–9)	9 (8–9)	***p*** **= 0.003**
VMT-learning	8 (8–9)	10 (9–10)	***p*** **< 0.001**
VMT-delayed recall	8 (6–9)	9 (8–10)	***p*** **< 0.001**
VMT-recognition	10 (10–10)	10 (10–10)	*p* = 0.135
Digit span forward	5 (4–5)	5 (4–6)	*p* = 0.75
Digit span backward	3 (2.5–4)	3 (3–4)	*p* = 0.89

*TBI, traumatic brain injury, MMSE, mini mental state examination; FAB, frontal assessment battery; VMT, visual memory test. Bold values indicate the comparisons that reach statistic significant differences, considering the significance level as p < 0.05*.

When compared to normative published data, 26.4% of patients showed deficit at MMSE and 9.4% at the FAB. In the Visual Memory Test, 17% exhibited deficits on naming, 3.8% (*n* = 2) on VMT incidental memory, 18.9% (*n* = 10) on VMT immediate recall, 22.6% (*n* = 12) on VMT learning, 13.2% (*n* = 7) on VMT delayed recall, and 7.5% (*n* = 4) at VMT recognition. There were no patients with deficits at the Digit Span test.

### History of Previous TBI

In order to investigate whether a history of previous TBI influenced cognitive performance, patients with previous TBI (TBI+) was compared to those without a previous history of TBI (TBI-). The comparison is shown in Table 3. These data were available for 51 out 53 participants. Twenty and eight patients (54.9%) composed the TBI+ group and 23 (45%) the TBI- group. The groups were similar regarding sex (*p* = 0.76), age (*p* = 0.23), and years of education (*p* = 0.78). No significant differences were found between groups regarding cognitive performance (Table 3).

**Table 3:** Cognitive performance comparison between patients with and without previous TBI history.

**Variables**	**Previous TBI – median (percentile 25–75)**	**No previous TBI – median (percentile 25–75)**	**Mann-Whitney test**
*N* (%)	28 (54.9%)	23 (45.1%)	–
MMSE	23.5 (21.25–26.75)	25.2 (21.5–26.25)	*p* = 0.45
FAB	12 (10.25–14.75)	12 (11–14)	*p* = 0.93
VMT-naming	10 (10–10)	10 (10–10)	*p* = 0.92
VMT-incidental memory	6 (5–7)	6 (5–7)	*p* = 0.75
VMT-immediate memory	8 (7–9)	8 (7–9)	*p* = 0.71
VMT-learning	8 (7–9)	8 (8–9)	*p* = 0.86
VMT-delayed recall	8 (6–9)	8 (6–9)	*p* = 0.77
VMT-recognition	10 (10–10)	10 (10–10)	*p* = 0.23
Digit span forward	4.5 (4–5.75)	4 (4–5)	*p* = 0.90
Digit span backward	3.5 (2.25–4)	3 (2–4)	*p* = 0.37

*TBI, traumatic brain injury; MMSE, mini mental state examination; FAB, frontal assessment battery; VMT, visual memory test*.

### Loss of Consciousness

In order to investigate whether loss of consciousness (LOC) influenced cognitive performance, patients who had LOC (LOC+) were compared to those without LOC (LOC–). The comparison is shown in Table 4. This information was available for 52 out 53 participants. Twenty and seven patients (51.9%) composed the LOC+ group and 25 (48%) the LOC– group. No significant differences regarding sex (*p* = 0.15) and years of education (*p* = 0.86) were found. There was a significant difference of age (*p* = 0.04; *d* = 0.588), with the LOC+ group (Mean = 43.2; *SD* = 13.6) being older than the LOC– group (Mean = 35.4; *SD* = 12.7). No significant difference was found between groups regarding cognitive performance (Table 4).

**Table 4:** Cognitive performance comparison between patients with and without loss of consciousness.

**Variables**	**LOC – median (percentile 25–75)**	**No LOC – median (percentile 25–75)**	**Mann-Whitney test**
*N* (%)	27 (51.9%)	25 (48.1%)	
MMSE	23 (20.75–26)	26 (22.5–27)	*p* = 0.16
FAB	12 (10–14)	13 (11.5–15)	*p* = 0.08
VMT-naming	10 (9–10)	10 (10–10)	*p* = 0.10
VMT-incidental memory	6 (4–7)	6 (5–6.75)	*p* = 0.88
VMT-immediate memory	8 (6–9)	8 (7–8)	*p* = 0.36
VMT-learning	8 (7–9)	8.5 (8–9.75)	*p* = 0.29
VMT-delayed recall	8 (6–9)	8 (7–9)	*p* = 0.48
VMT-recognition	10 (10–10)	10 (10–10)	*p* = 0.25
Digit span forward	5 (4–5)	4 (4–5.5)	*p* = 0.90
Digit Span backward	3 (2–4)	3 (2.5–4)	*p* = 0.83

*TBI, traumatic brain injury; MMSE, mini mental state examination; FAB, frontal assessment battery; VMT, visual memory test*.

### Years of Education

To investigate a potential protective role of years of education in mTBI-associated cognitive deficits, patients were divided into two groups and their scores compared. Twenty-nine patients (54.7%) had accomplished basic education or less (until 8 years of formal education) and 24 patients (45.3%) had accomplished more than 8 years of education. No significant differences regarding sex (*p* = 0.56) and age (*p* = 0.23) were observed. Significant differences were found in MMSE (≤8 years – median = 22; >8 years – median = 26; *p* < 0.01; *d* = 1.115), FAB (≤8 years – median = 12; >8 years – median = 14; *p* < 0.01; *d* = 1.094), Digit Span forward (≤8 years – median = 4; >8 years – median = 5; *p* < 0.01; *d* = 0.732) and Digit Span backward (≤8 years – median = 3; >8 years – median = 4; *p* < 0.02; *d* = 0.669). Differences were also significant in VMT naming (≤8 years – median = 10; >8 years – median = 10; *p* < 0.02; *d* = 0.672), VMT immediate memory (≤8 years – median = 7; >8 years – median = 8; *p* < 0.03; *d* = 0.638) and VMT learning (≤8 years – median = 8; >8 years – median = 8.5; *p* < 0.049; *d* = 0.567). No differences were found in VMT incidental memory (*p* < 0.26), VMT recall (*p* < 0.12), and VMT recognition (*p* < 0.38).

The frequency of impairment (i.e., more than 2 standard deviations below mean) between these two groups were also compared (Table 5). Significant differences were found between groups in VMT naming (*p* < 0.02; *d* = 0.679), VMT immediate memory (*p* < 0.01; *d* = 0.756) and VMT learning (*p* < 0.01; *d* = 0.913), with increased frequency of impairment in the group with ≤ 8 years of education.

**Table 5:** Frequencies of cognitive deficits among mTBI patients according to education.

**Neuropsychological tests**	**Impairment**	**≤8 years of education – *N* (%)**	**>8 years of eduacation**	**Total – *N***	**Pearson Chi-square**
MEEM	No	19	19	38	*p* = 0.17
	Yes	10 (34.5%)	4 (17.4%)	14	
FAB	No	25	23	48	*p* = 0.23
	Yes	4 (13.8%)	1 (4.2%)	5	
VMT naming	No	20	23	43	***p*** **= 0.02**
	Yes	8 (28.6%)	1 (4.2%)	9	
VMT incidental memory	No	27	23	50	*p* = 0.91
	Yes	1 (3.6%)	1 (4.2%)	2	
VMT immediate memory	No	19	23	42	***p*** **= 0.01**
	Yes	9 (32.1%)	1 (4.2%)	10	
VMT learning	No	17	23	40	***p*** **= 0.003**
	Yes	11 (39.3%)	1 (4.2%)	12	
VMT delayed recall	No	22	23	45	*p* = 0.07
	Yes	6 (21.4%)	1 (4.2%)	7	
VMT recognition	No	25	23	48	*p* = 0.38
	Yes	3 (10.7%)	1 (4.2%)	4	

*MMSE, mini mental state examination; FAB, frontal assessment battery; VMT, visual memory test. Bold values indicate the comparisons that reach statistic significant differences, considering the significance level as *p* < 0.05*.

### Correlations

We explored correlations between clinical variables (age, GCS, alcohol, and drugs) and neuropsychological tests among patients. Significant negative correlation was observed between age and VMT naming (*r* = −0.308; *p* < 0.02). GCS was positively correlated with FAB (*r* = 0.354; *p* < 0.009) and negatively with alcohol use (*r* = −0.372; *p* < 0.006).

As there was a difference of age between patients who had LOC and those who had not was found, we investigated whether age and LOC were correlated. Age significantly correlated with LOC (*r* = 0.285; *p* = 0.04). Moreover, when control group was included in the analysis of age and naming, the correlation remained significant (*r* = −237, *p* = 0.03).

## Discussion

To the best of our knowledge, this is the first study to investigate the incidence of cognitive impairment following acute mTBI in a Brazilian population and to identify factors that may play a protective role at the acute phase. Previous Brazilian studies evaluated TBI-related cognitive deficits only in its chronic phase and with different severity (27–29).

We found cognitive dysfunction in a significant percentage of people with mTBI, especially in general cognitive ability (26.4%), learning memory (22.6%), and immediate memory (18.9%). Also, compared to a healthy control group, mTBI patients showed significantly worse cognitive performance on general cognitive ability, naming, incidental memory, immediate memory, learning memory, delayed recall, and executive functioning. Our findings are in agreement with previous studies that reported significant cognitive deficits in the acute phase of post-TBI, mainly associated with episodic memory (4–9). A meta-analysis also reported significant impairment in verbal and visual memories immediately after mTBI (30), thus supporting our data. Moreover, compared with healthy controls, patients with mTBI performed significantly worse in attention, memory, language and executive functions at acute phase of TBI and at 6 month follow-up (4). Neuropsychological measures at the acute phase were also significantly associated with changes in white matter integrity in brain regions such as the splenium of corpus callosum and cingulum (4). A more recent study showed impairment in selective attention/inhibitory control, divided attention, and working memory in acute mTBI (<7 days) (31). These deficits were associated with gray matter morphological changes (31). Taken together, these studies reinforce our findings by providing evidence of impairment in several cognitive domains in the acute phase of mTBI.

It has been reported that LOC might be associated with increased cognitive deficits following mTBI. For instance, military service members with blast-related mTBI with LOC presented severe memory impairment, sleep disorders, post-traumatic symptoms, and slow simple reaction times at 72 h post-brain injury (13). LOC was also associated with avoidance symptoms, worse quality of life and post-concussion symptoms in veterans with mTBI and comorbid PTSD (32). LOC might also influence cognitive outcome since uncomplicated mTBI patients without LOC presented early recovery compared with those who had LOC at 45 days following the TBI event (12). Patients who presented mTBI with LOC were more likely to present deficits in executive functions and associated disruption in white matter integrity in ventral prefrontal areas (14). We did not find significant differences in cognitive performance between mTBI patients with and without LOC at 24 h post-brain injury. Methodological differences, including an early time point evaluation (24 h post-mTBI) and the cognitive tests applied, might explain, at least in part, these results. In our study, we used mainly screening tests, while the above-mentioned studies used computerized tests or a more comprehensive neuropsychological assessment, which are more sensitive.

Similarly to LOC, we did not find any difference in the cognitive performance between patients with and without history of previous TBI. Our findings are in agreement with Comerford et al. (6) who reported that mTBI patients with history of previous TBI did not show lower cognitive performance than those without history. On the other hand, repetitive mTBIs have been associated with a worse outcome than a single, uncomplicated mTBI, often leading to long-term cognitive impairment and persistent post-concussive syndrome, which also includes somatic and emotional symptoms (15, 30, 33–36). It has been postulated that a single mTBI, without findings on brain scan, has a complete resolution after 3 months (15, 30, 35). However, this does not seem to be true for all mTBI cases since cognitive and behavioral changes have been reported longer than 3 months after a single traumatic event (10, 37). Similarly to the LOC results, these controversial findings might also be at least in part explained by different methodological approaches.

Years of education seem to influence cognitive performance following acute mTBI since patients with 8 years or less of education showed a higher frequency of deficits in the cognitive tests compared with those with higher educational levels. These findings support a protective role of education in cognitive decline associated with mTBI. Similar to our findings, Sumowski et al. (38) found that higher educational level attenuated the deleterious effects of TBI. Individuals with moderate to severe TBI and higher educational attainment performed better in cognitive tests than TBI patients with low educational attainment. As observed by Leary et al. (39), years of education were associated with measures of memory, learning, working memory and processing speed in patients with TBI. Education is one of the factors that constitute the so-called “cognitive reserve,” a protective factor associated with better TBI outcome. Indeed, a meta-analysis showed that higher education and higher pre-morbid IQ were found to be associated with better outcomes following TBI (40). Conversely, lower levels of cognitive reserve, as measured by educational attainment, premorbid IQ and occupational skill level, were associated with worse recovery, higher risk for post-concussive symptoms in mTBI patients 3 months after injury (41). Together, these findings support that education is a protective factor against TBI, and our findings indicate that education may be a protective factor at early stages of mTBI, as in the first 24 h.

It is worth mentioning that other characteristics such as baseline GCS may also influence TBI-associated cognitive impairment. Dikmen et al. (36) found that patients with GCS of 15 and abnormal findings on CT presented significant impairment in episodic memory and functional outcome. Cognitive performance was even more impaired in patients with GCS 13-14 and abnormal CT findings, with significant deficits in episodic memory, attention, inhibitory control, cognitive flexibility, processing speed, motor performance, verbal intelligence, and functional outcome (36). In the current study, the GCS was 15 for the majority of the patients (92.4%) and those with abnormal findings on CT were excluded, suggesting that other features like years of education might be taken into account in order to better characterize cognitive functions in acute mTBI population.

In the current study, a positive correlation between GCS and FAB score was found along with a positive association between GCS and alcohol use report. In accordance with our findings, Yue et al. (42) showed an association between higher blood alcohol levels and lower score in the GCS at the acute stage in patients with uncomplicated mTBI. Blood alcohol levels were also associated with prolonged LOC. Excessive alcohol consumption was associated with decreased non-verbal processing speed and worse recovery at 6 months after injury. Contrary to our findings, Rojas et al. (43) found no correlation between GCS and FAB score when assessing patients with TBI at the acute stage. However, they found that the combined GCS and FAB scores can be used as predictors of TBI outcome at the acute stage. This difference may be accounted by differences in sample, since this latter study evaluated mild and moderate TBI and observed no difference of performance at FAB between those groups.

In our sample, mTBI patients performed significantly worse than healthy controls on naming, and naming correlated negatively with age. In agreement to our findings, Gauthier et al. (44) observed that patients with mTBI performed significantly worse than controls on naming when evaluated within 2 weeks post-TBI. Also, they found that age predicted performance on naming, with older age being associated with worse naming scores. It is well known that aging is associated with name retrieval difficulties, that may not be apparent in the speech, in which the subject can use similar words to replace the forgotten one, but do appear in naming tasks, where the answer is more restricted (45). Naming may be affected not only by age, but by low education, that can interfere in naming tasks (46).

Interestingly, a significant difference in age between patients with and without LOC was found. Moreover, age and LOC also correlated positively. Accordingly, LOC has been pointed out as a potential predictor of intracranial lesion in older patients with mTBI (47), supporting the hypothesis that older age might be associated with poorer outcomes. Brain imaging and gene expression studies also revealed that aging is associated with worse neural recovery in response to an acute mTBI (48).

Our study has limitations that must be considered. Other factors not evaluated in our study may have influenced cognitive performance, such as psychological stress, premorbid psychiatric status and previous cognitive decline (49, 50). Future studies must control these factors.

In sum, we found that patients with mTBI present cognitive deficits at acute stage (<24 h), mainly in episodic memory and executive function. Factors such as LOC and previous TBI seem to have minor influence during this period. On the other hand, factors such age and years of education may influence cognition, with younger ages and higher education level playing protective roles.

## Minas Gerais' Traumatic Brain Injury Study Group

Ananda Peixoto Silva; Antônio Bernardes Bacilar Campos, Camila Carvalhais Costa, Christian Pereira Antônio, Daniela Lanna e Melo Loures, Eduarda Félix Ponte, Felipe Vieira Guarçoni, Guilherme Ribeiro Mansur Barbosa, Ilanna Naoli Santos Miranda, Jordana Campos Queiroz, Leonardo Gomes Salomão, Letícia Monteiro de Souza Oliveira, Maria Cecília Landim Nassif, Mariana Braga Valadão, Millena Vieira Brandão Moura, Otávio Fonseca Rodrigues, Pedro Henrique Lodde Leal, Tatiana Costa Diamantino, Letícia Siqueira Araújo, Thiago de Oliveira Furlan, Ewelin Wasner Machado da Silva, Isadora Gonçalves Roque, Alessandra Noronha da Silva.

## Author Contributions

MGFC in the experimental design, carried out neuropsychological assays, data analysis, and drafted the manuscript. RMF participated in the study design and coordination and revised and edited the manuscript. JJP participated in the study design and neuropsychological data analysis and revised the manuscript. AK participated in the study design, data analysis and edited the manuscript. PC was responsible for analysis and interpretation of data and revised and edited the manuscript. ALT participated in the design and coordination of the study, revised, and edited the manuscript. LCS and ASM designed and coordinated the study, were responsible for data interpretation, revised, and edited the manuscript. All authors have read and approved the final version of the manuscript.

### Conflict of Interest Statement

The authors declare that the research was conducted in the absence of any commercial or financial relationships that could be construed as a potential conflict of interest.
